# Identification of the shared hub gene signatures and molecular mechanisms between HIV-1 and pulmonary arterial hypertension

**DOI:** 10.1038/s41598-024-55645-x

**Published:** 2024-03-25

**Authors:** Huanzhuo Mai, Xing Yang, Yulan Xie, Jie Zhou, Yiru Wei, Tingyan Luo, Jing Yang, Ping Cui, Li Ye, Hao Liang, Jiegang Huang

**Affiliations:** 1 School of Public Health, Guangxi Medical University, Nanning, 530021 China; 2 Guangxi Key Laboratory of AIDS Prevention and Treatment, Guangxi Medical University, Nanning, 530021 China; 3 Guangxi Academy of Medical Sciences, The People’s Hospital of Guangxi Zhuang Autonomous Region, Nanning, 530021 China; 4 Life Science Institute, Guangxi Medical University, Nanning, 530021 China; 5 Guangxi Colleges and Universities Key Laboratory of Prevention and Control of Highly Prevalent Diseases, Guangxi Medical University, Nanning, 530021 China

**Keywords:** HIV-1, Pulmonary arterial hypertension, Type I IFN, Hub genes, scRNA-seq, HIV infections, Cardiovascular diseases

## Abstract

The close link between HIV-1 infection and the occurrence of pulmonary arterial hypertension (PAH). However, the underlying molecular mechanisms of their interrelation remain unclear. The microarray data of HIV-1 and PAH were downloaded from GEO database. We utilized WGCNA to identify shared genes between HIV-1 and PAH, followed by conducting GO and pathway enrichment analyses. Subsequently, differentially genes analysis was performed using external validation datasets to further filter hub genes. Immunoinfiltration analysis was performed using CIBERSORT. Finally, hub gene expression was validated using scRNA-seq data. We identified 109 shared genes through WGCNA, primarily enriched in type I interferon (IFN) pathways. By taking the intersection of WGCNA important module genes and DEGs, ISG15 and IFI27 were identified as pivotal hub genes. Immunoinfiltration analysis and scRNA-seq results indicated the significant role of monocytes in the shared molecular mechanisms of HIV-1 and PAH. In summary, our study illustrated the possible mechanism of PAH secondary to HIV-1 and showed that the heightened IFN response in HIV-1 might be a crucial susceptibility factor for PAH, with monocytes being pivotal cells involved in the type I IFN response pathway. This provides potential new insights for further investigating the molecular mechanisms connecting HIV-1 and PAH.

## Introduction

Acquired immunodeficiency syndrome (AIDS) is a fatal infectious disease that significantly damages the human immune system andmainly caused by infection with the human immunodeficiency virus type 1 (HIV-1)^1^. Although anti-retroviral therapy (ART) can significantly reduce the mortality of HIV-1/AIDS patients^2^, 6.5 million people still died from HIV-1/AIDS related causes worldwide over the past period^3^. With the widespread application of ART, the survival period of HIV-1 infected individuals has significantly extended. However, there is still a significant disparity between those infected with HIV-1 and the general population in terms of life expectancy and quality of life, which can largely be attributed to complications caused by HIV-1 infection^4^. As survival time of HIV-1 infections, the incidence of chronic complications associated with HIV-1 has significantly increased^5,6^, particularly chronic lung disease^7^ and cardiovascular disease^8^.

Pulmonary arterial hypertension (PAH) as a cardiovascular disease with high morbidity and mortality^9^. The main characteristic of PAH is an elevated pulmonary arterial pressure (PAP). PAH is caused by hyperproliferation of pulmonary arterial smooth muscle cells, apoptosis, and metabolism^10^, resulting in an abnormally elevated PAP and pulmonary vascular resistance (PVR), which may lead to progressive right heart failure and ultimately contribute to patient mortality, making PVR a major cause of death in individuals with PAH^11,12^. HIV-1 infection is considered to be one of the major causes of pulmonary arterial hypertension, triggering dysregulation of inflammatory and immune responses as well as endothelial dysfunction^13^, which serves as one of the crucial pathogenic mechanisms of PAH^14^. However, the etiology and pathogenesis of PAH remain incompletely elucidated, particularly in cases associated with HIV-1. Furthermore, the survival rate of patients with HIV-1-related PAH is half of that of HIV-1 infected individuals without PAH^15^. Despite epidemiological studies showing a strong positive relationship between HIV-1 infection and the occurrence of PAH, several studies have demonstrated that HIV-1 does not directly alter the pathogenic mechanisms of lung disorders, including PAH^16^. Consequently, the mechanism underlying the relationship between HIV-1 infection and PAH still requires further exploration.

As gene microarray and scRNA-seq technology continue to advance and mature to facilitate, unraveling of the genetic and cellular intricacies of various diseases, we are prompted to explore novel frontiers in the realm of gene vaccines. Simultaneously, a comprehensive investigation into the relationship between HIV-1 and PAH not only enhances our understanding of shared pathophysiological features but also opens new prospects for the development of gene vaccines. The potential of gene vaccines lies in their ability to leverage the genetic information of pathogens to stimulate a targeted and robust immune response. In the context of HIV-1 and PAH, delving into shared molecular mechanisms may provide crucial insights which allow gene vaccines to be designed that are tailored to address both challenges.

In this study, we conducted bioinformatics analyses using gene expression profiles from public databases to identify shared hub genes and pathways in HIV-1 and PAH patients. We also investigated the correlation between hub genes and immune cells. We further explored the expression patterns of hub genes in various subpopulations of cells using single-cell RNA sequencing (scRNA-seq) data. This may be the first study to explore the hub gene features shared between HIV-1 and PAH. We aim to better understand their intricate interplay and shared molecular mechanisms; outline prospects for guiding and optimizing vaccine development; and provide valuable insights for designing targeted and effective immune strategies.

## Materials and methods

### datasets acquisition and processing

We performed a comprehensive search of the Gene Expression Omnibus (GEO) database (https://www.ncbi.nlm.nih.gov/geo/) and ArrayExpress (https://www.ebi.ac.uk/arrayexpress/) using the key terms “human immunodeficiency virus” or “pulmonary arterial hypertension” to find the gene expression profiles of HIV-1 and PAH patients. Datasets were selected based on the following criteria: (1) the gene expression profiling contain cases and controls. (2) Gene expression data were obtained using array gene expression chips. (3) The sample type used for sequencing is peripheral blood mononuclear cell (PBMC). (4) At least 3 independent samples per group were used for gene expression analysis. (5) The raw data is required for gene expression profiling to facilitate re-analysis. Based on the above selection criteria, a total of six datasets were ultimately included in this study, consisting of three datasets related to HIV-1 (GSE140713, GSE77939 and GSE2171) and three datasets related to PAH (GSE33463, GSE703 and GSE19617). Comprehensive details of the information of included studies are provided in Table 1, including GSE numbers, detection platforms, types of RNA sources, and sample information. The raw expression arrays from the Affymetrix platform were background corrected using robust multi-array average (RMA) algorithm through the affy package^17^, and the raw expression arrays from the Agilent and Illumina platforms were quantile-normalized and log2 transformed through the limma package^18^. The probes were matched to gene symbols according to the annotation information of the corresponding platform, and those without the corresponding gene symbol were removed. The gene expressions were averaged when multiple probes were matched to the same gene symbols. Principal component analysis (PCA) was performed to visualize the spatial distribution of samples.

**Table 1:** Study characteristics of the included studies.

GEO accession number	Disease	Organization type	PMID	Platform	Sample information
GSE140713	HIV-infected	PBMC	34871208	GPL6480	HIV = 50 (All HIV patients were received antiretroviral therapy); control = 7
GSE77939	HIV-infected	PBMC	32579550	GPL15207	HIV = 17 (included 7 patients with ART-IF, 5 patients with ART-N and 5 patients with ART-R); control = 4
GSE2171	HIV-infected	PBMC	15897992	GPL201	HIV = 22 (HIV patients did not receive ART); control = 12
GSE33463	PAH	PBMC	22545094	GPL6947	PAH = 72 (included 30 patients with IPAH and 42 patients with SPAH); control = 41
GSE703	PAH	PBMC	15215156	GPL80	PAH = 14 (included 4 patients with IPAH and 10 patients with SPAH); control = 6
GSE19617	PAH	PBMC	20808962	GPL6480	PAH = 15 (15 patients with SPAH); control = 10

*ART-IF* antiretroviral therapy immunolgical failure (individuals undergoing ART for at least 1 year, exhibiting an undetectable viral load (< 40 copies/mL), and maintaining CD4 T cell counts below 250 cells/μL), *ART-N* antiretroviral therapy naive (ART-naive individuals with a CD4 T cell count exceeding 200 cells/μL), *ART-R* antiretroviral therapy responder (Individuals undergoing ART for at least 1 year, showing a positive response to the treatment by exhibiting an increase in CD4 T cell counts of over 150 cells/μL, with current CD4 counts above 250 cells/μL), *IPAH* idiopathic pulmonary arterial hypertension, *SPHA* secondary pulmonary arterial hypertension.

### Weighted gene coexpression network analysis and identification of shared genes in HIV-1 and PAH

Weighted correlation network analysis (WGCNA) can be used to find the co-expressed gene modules with high biological significance, construct the gene coexpression network, and identify the modules correlated to diseases^19^. According to the WGCNA tutorial, which recommends a sample size of more than 20 for robust results, we selected datasets with relatively large sample sizes (GSE140713 and GSE33463) for WGCNA analysis. Firstly, we selected the top 25% of the genes with the largest variance in the expression matrix, including about 5000 genes for the WGCNA analysis. Then we utilized the goodSamplesGenes function in the WGCNA package to filter for samples and genes with excessive missing values and zero variation, and remove outliers by sample hierarchical clustering with hclust function in the WGCNA package. Based on the criteria of approximate a scale-free topology (R^2^ > 0.85) and mean connectivity (approach to 0), we select the best soft threshold value (β) from a range of 1 to 20, using the PickSoftThreshold function in the WGCNA package. In this study's WGCNA analysis, the soft threshold for GSE140713 was 14 and the soft threshold for GSE33463 was 6. Subsequently, the adjacency matrix was transformed into a topological overlap matrix (TOM) and a hierarchical clustering dendrogram was constructed. Utilizing the blockwiseModules function from the WGCNA package, we categorized similar gene expressions into different modules with the major parameters: corType = “pearson”, networkType = “unsigned”, minModuleSize = 20, mergeCutHeight = 0.25. Following the construction of the co-expression network, the module eigengenes were calculated using the moduleEigengenes function in the WGCNA package and performed correlation analysis with phenotypic traits.

### Identification of shared gene features in HIV-1 and PAH

We selected modules with high correlation coefficients (r ≥ 0.5 and *P* < 0.05) associated with HIV-1 and PAH, and identified overlapping shared genes in moudulethrough an online Venn diagram tool (http://www.interactivenn.net/). To explore the potential roles of shared genes in HIV-1 and PAH, biological analysis was performed through ClueGO (version 2.5.9), a Cytoscape software (version 3.9.1) plugin that categorizes non-redundant Gene Ontology (GO) terms and visualizes them as a network of functional groups^20^. Significant biological processes (BP) were identified through Gene Ontology (GO) analysis, with a significance threshold of *P* < 0.05.

### Identification of hub genes through differentially expressed genes analysis and WGCNA

Differential expression analysis of the GSE77939 and GSE703 datasets was conducted using the voom method^21^ via the limma package. Differentially expressed genes (DEGs) between HIV-1 and PAH were determined to compare control group and HIV-1/PAH group gene expression profiling for subsequent analysis. The screening criteria for DEGs in both the GSE77939 and GSE703 datasets were |log2 fold change (FC)|> 0.585 and *P* < 0.05. By overlapping DEGs positively correlated with HIV-1/PAH and genes shared from WGCNA-related modules, we ultimately identified hub genes through a Venn diagram.

### Hub genes expression levels and diagnostic potential in HIV-1 and PAH

The Student's t-test was used to compare the expression levels of hub gene between the control group and the HIV-1/PAH group. Considering the impact of ART effectiveness in HIV patients and the potential influence of different subgroups within PAH classifications on the results, we conducted subgroup comparative analyses separately. The area under the curve (AUC) of the receiver operating characteristic (ROC) curve was used to evaluate the prediction effectiveness of potential biomarkers on the datasets (GSE140713, GSE77939, GSE2171, GSE33463, GSE703 and GSE19617) via the pROC package^22^.

### Construction of protein–protein interaction network for hub genes and conducting gene ontology and pathway enrichment analyses

In order to explore the mechanisms underlying the functions of hub genes, we utilized the GeneMania database (http://www.genemania.org) to identify proteins that are functionally associated with the hub genes and constructed the corresponding protein–protein interaction (PPI) network. Subsequently, gene ontology (GO) and pathway enrichment analysis for these associated genes was performed using the Metascape online platform (https://metascape.org).

### Gene set enrichment analysis

Gene set enrichment analysis (GESA) is an enrichment analytical method for interpreting gene expression data that calculates an enrichment score to determine whether a pre-defined gene set displays statistically significant differences between two biological states^23^. Based on the median cutoff values of ISG15 and IFI27 expression in the GSE140713 and GSE33463 datasets, we categorized HIV-1/PAH patients into groups with high and low expression levels of ISG15, as well as high and low expression levels of IFI27.DEGs analysis was performed using the limma package, GSEA was performed using the clusterProfiler package^24^ to identify the hallmark gene sets from the Human Molecular Signatures Database (MSigDB v7.4, https://www.gsea-msigdb.org/gsea/msigdb/). All GSEA enrichment plots were generated with the GseaVis package (https://github.com/junjunlab/GseaVis). Adjusted *P* < 0.05 (Benjamini–Hochberg correction) was considered significant. Significantly enriched pathways were screened based on normalized enrichment score (NES) ranking.

### Immune cell infiltration analysis

CIBERSORT is an anti-volume algorithm based on a gene expression matrix used to calculate the relative abundance of 22 immune cell types, including naive B cells, memory B cells, plasma cells, CD8 T cells, naive CD4 T cells, resting menmory CD4 T cells, activated memory CD4 T cells, T follicular helper cells, regulatory T cells, gamma delta T cells, resting natural killer (NK) cells, activated NK cells, monocytes, macrophages (M0), type 1 macrophages (M1), type 2 macrophages (M2), activated dendritic cells (DCs), resting DCs, activated mast cells, resting mast cells, eosinophils, neutrophils. We performed the immune cell infiltration analysis of the GSE140713 (HIV) and GSE33463 (PAH) datasets via the cibersort package and filtered immune-cell types with an abundance of 0. The Wilcoxon rank sum test was used to compare the immune cell infiltration between the control group and the HIV-1/PAH group, and the correlation between hub genes and different immune cell types was determined via Spearman correlation analysis. The correlation coefficient |cor|> 0.3 and *P* < 0.05 was considered with statistical significance.

### Single-cell dataset processing and clustering analysis

Single-cell transcriptome matrix datasets were obtained using the 10X Genomics platform, including one sourced directly from 10X Genomics (8381 PBMCs) and two others acquired from the GEO database (GSE157829 and GSM6647828). Seurat objects were created for each dataset using the Seurat package (version 4.3.0), and doublets were predicted and removed using the DoubletFinder package (version 2.0.3) according to the 10× Genomics Chromium Single Cell 3′ Reagent Kits User Guide (v2 Chemistry). Subsequently, we filtered out cells with under 200 expressing genes, over 5000 expressing genes and over 15% mitochondrial gene expression. Following this filtering step, the filtered datasets were individually normalized and scaled using SCTransform. The cell cycle scores based on S and G2M phase markers were calculated by CellCycleScoring function in the Seurat package. The integrated analysis of these datasets was conducted using the canonical correlation analysis (CCA) method. Afterward, integrated data were then clustered and visualized using the top 20 principal components by the generated Elbow plot (Fig. S1). Clustering was performed via the FindNeighbors and FindClusters functions in Seurat with a resolution of 0.8. Marker genes for each cluster were identified with the FindAllMarkers function from Seurat with parameters min.pct = 0.25 and logfc.threshold = 0.25. We identified major cell clusters present in the healthy and HIV-1 infected donors based on expression of marker genes signature: T cells (CD3D, CD8B, IL7R, CCR7, CD27), NK cells (GNLY, NKG7, GZMB), B cells (MS4A1), monocytes (LYZ, S100A9, CD14, FCGR3A), Megakaryocytes (PPBP) and plasmacytoid dendritic cell (pDCs; LYZ, IGJ). Furthermore, we employed the AUCell package (v1.12.0) to calculate the gene expression signature score for the crucial hallmark pathway.

### Statistical analysis

All statistical analyses and visualization were performed with R Studio (Version 4.2.1; R studio Inc., Boston, MA, USA). The *P* < 0.05 was considered significant.

## Results

**Figure 1:**
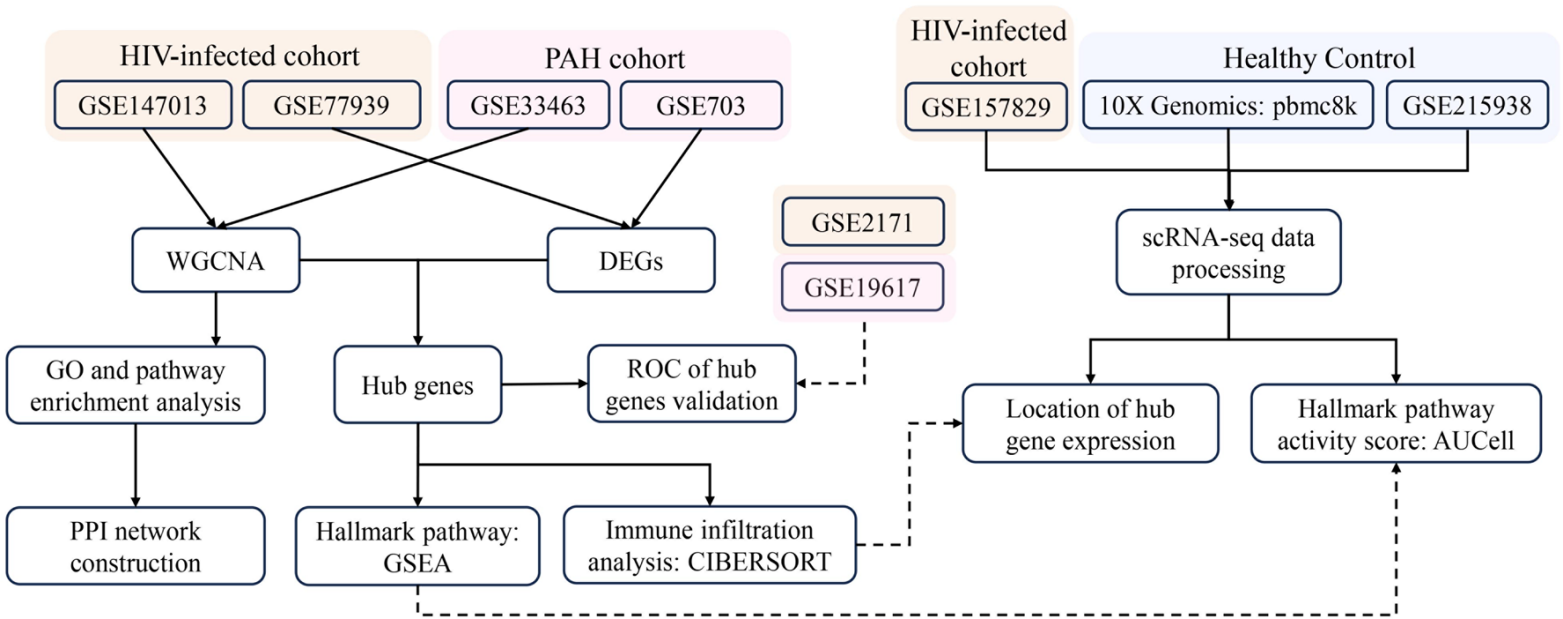
The comprehensive workflow diagram of this study.

The flow chart of the overall design in this study is shown in Fig. 1. We performed quality controls on the six datasets (GSE140713, GSE77939, GSE2171, GSE33463, GSE703 and GSE19617) included in this study, and the box diagram of microarray signal intensity confirmed that the data was of good quality (Fig. S2).

### Construction of the WGCNA network and identification of crucial modules

**Figure 2:**
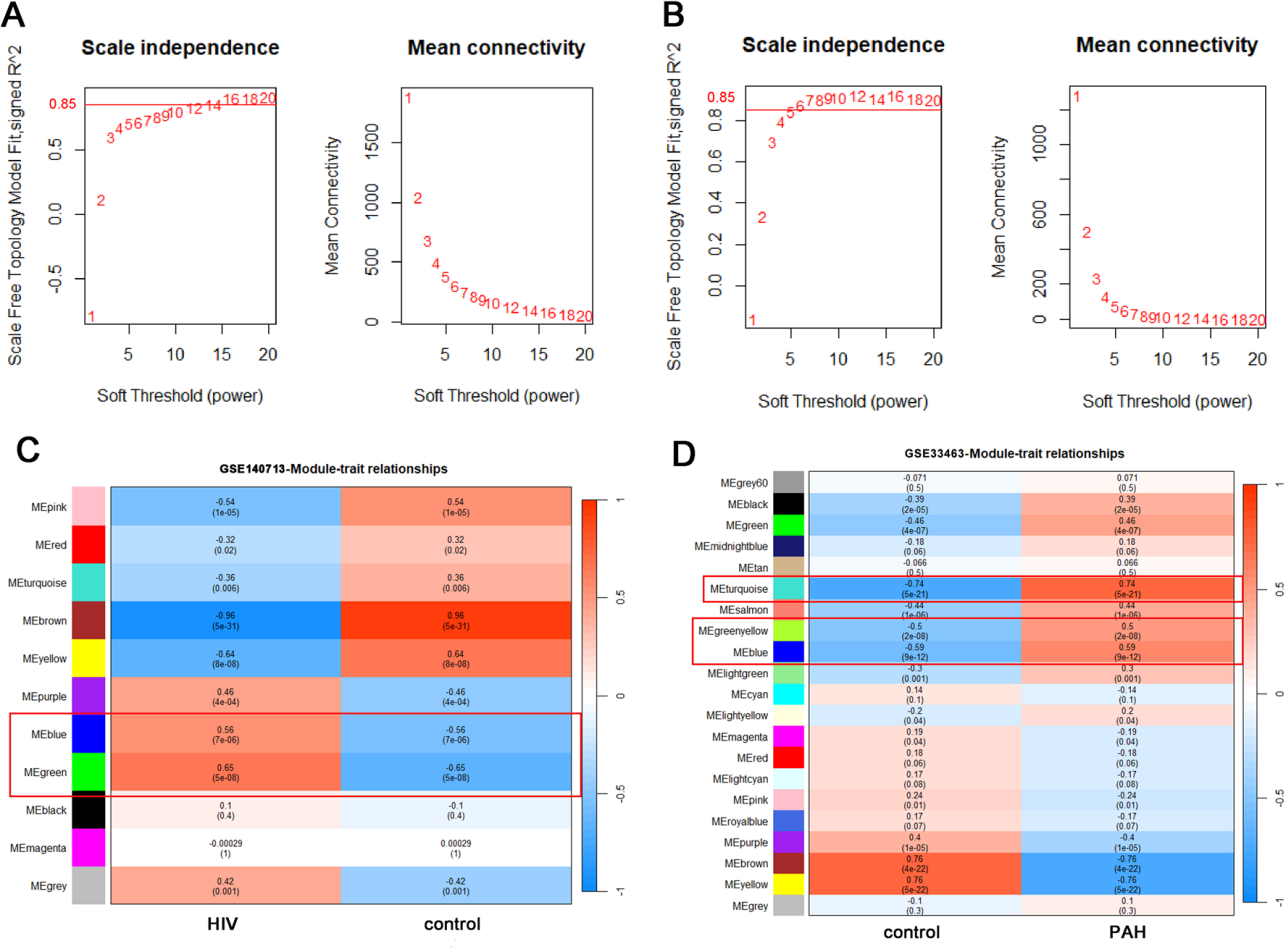
Weighted gene co-expression network analysis (WGCNA) of GSE140713 (HIV) and GSE33463 (PAH). (**A**) Scale-free network analysis under different soft-thresholding powers for GSE19187 (HIV). The left side shows the scale-free topology fit index (R2) across a range of soft threshold powers (β) from 1 to 20. On the right side, the mean connectivity is displayed for each β value from 1 to 20. (**B**) Scale-free network analysis under different soft-thresholding powers for GSE33463 (PAH). The left side shows the R^2^ across a range of β from 1 to 20. On the right side, the mean connectivity is displayed for each β value from 1 to 20. (**C**) Module–trait relationships in GSE19187 (HIV). Each cell contains the corresponding correlation and P value. (**D**) Module–trait relationships in GSE33463 (PAH). Each cell contains the corresponding correlation and p value. *PAH* pulmonary arterial hypertension.

We selected the GSE140713 and GSE33463 datasets for WGCNA and examined the abnormal values of the two data sets by sample clustering, as depicted in Fig. S3 showing the sample dendrograms and trait heatmaps, along with the cluster dendrograms. GSE33463 removed one outlier sample (Fig. S4). In order to ensure that the network conforms to the scale-free network, the β-value was established at 14 for GSE140713 and at 6 for GSE33463 (Fig. 2A,B). We identified associations between module genes and clinical phenotypes using the spearman-related coefficient (Fig. 2C,D). A total of 11 modules were identified in the network created by GSE140713, of which the green module (r = 0.65, *P* < 0.001, Fig. 2C) and the blue module (r = 0.56, *P* < 0.001) had strong positive correlations with HIV-1. Similarly, in the network created by GSE33463, 21 modules were identified, of which the turquoise module (r = 0.74, *P* < 0.001, Fig. 2D), the blue module (r = 0.59, *P* < 0.001), and the greenyellow module (r = 0.50, *P* < 0.001) had strong positive correlations with PAH.

### The shared gene features in HIV-1 and PAH

**Figure 3:**
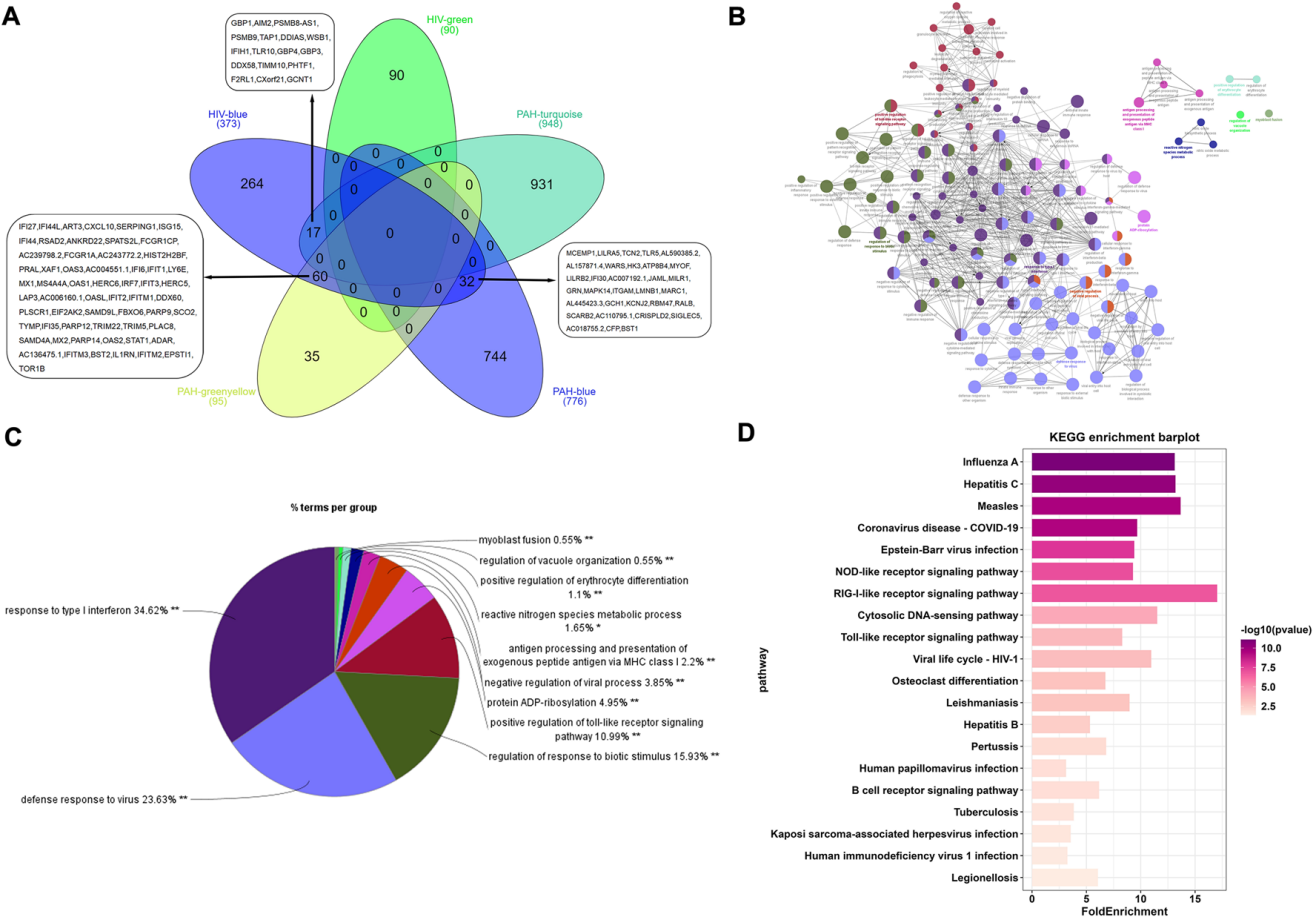
Identify shared genes between HIV and PAH, construct a protein–protein interaction (PPI) network, and perform Gene Ontology (GO) and pathway enrichment analysis. (**A**) The shared genes between the blue and green modules of HIV and greenyellow, blue and turquoise modules of PAH by overlapping them. (**B**) The interactive network of shared genes and their associated GO terms generated by the ClueGO plugin. The significant term of each group is highlighted. (**C**) The percentage of GO terms in the shared genes. (**D**) Top 20 KEGG pathway enrichment analysis results for the shared genes. *PAH* pulmonary arterial hypertension, *GO* Gene Ontology; *P < 0.05; **P < 0.01.

A total of 109 shared genes were identified that overlapped between modules positively associated with HIV-1 and PAH (Fig. 3A). Based on the interaction relationships among these 109 shared genes, we constructed a PPI network (Fig. 3B), and GO-BP enrichment analysis was performed on these genes via the ClueGO plugin. GO-BP enrichment analysis showed that those genes were mainly enriched in response to type 1 infection (IFN), the defense response to virus, regulation of response to biotic stimulus, and positive regulation of the toll-like receptor signaling pathway (Fig. 3C). According to KEGG enrichment analysis, those genes were primarily enriched for disorders caused by viral infections, the NOD-like receptor signaling pathway, and the RIG-I-like receptor signaling pathway (Fig. 3D).

### Identifying crucial hub genes through validating set of DEGs and WGCNA gene set

**Figure 4:**
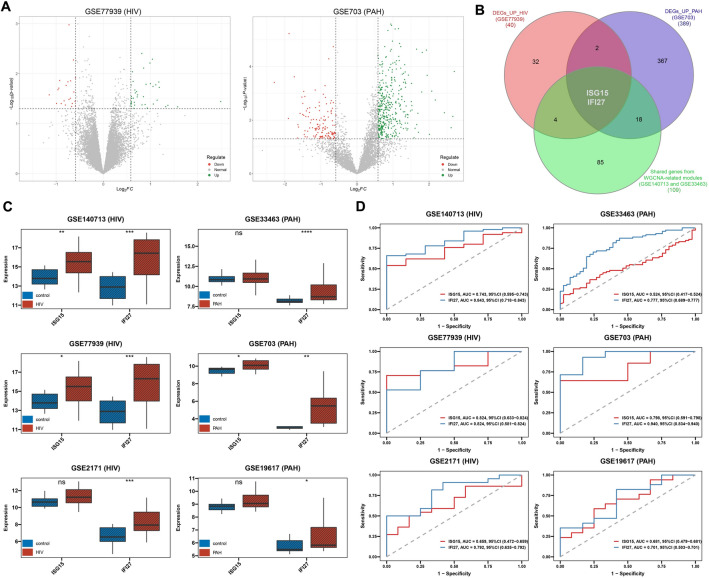
Identifying hub genes through differentially expressed genes (DEGs) and WGCNA, and assessing their diagnostic potential. (**A**) The left-side volcano plot showed 61 DEGs between the HIV patients and controls in GSE77939 (HIV). The right-side volcano plot showed 519 DEGs between the PAH patients and controls in GSE703 (PAH). Color represents the log2FC (red represents down-regulation, green represents up-regulation, and grey represents no change). (**B**) The Venn diagram showed the identification of hub genes by overlapping between upregulated DEGs identified in both HIV-1 (GSE77939) and PAH (GSE703) and co-expressed gene modules identified through WGCNA. (**C**) Expression of hub genes in different datasets. (**D**) ROC curve of the hub genes in different datasets. *PAH* pulmonary arterial hypertension, *FC* fold change, *WGCNA* weighted gene co-expression network analysis, *ROC* receiver operating characteristic; *P < 0.05; **P < 0.01; ***P < 0.001; ****P < 0.0001.

After the normalization of the datasets, we performed a principal component analysis (PCA). There were significant differences between the two groups (Fig. S5). We subsequently performed differential gene expression analysis for the GSE77939 and GSE703 datasets (Fig. 4A). A total of 61 DEGs, including 40 upregulated and 21 downregulated genes, were identified in the GSE77939 dataset. Similarly, in the GSE703 dataset, a total of 519 DEGs were identified, including 389 upregulated and 130 downregulated genes. We performed an intersection analysis between these upregulated genes and the WGCNA gene set using a Venn diagram, which identified the crucial hub genes as ISG15 and IFI27 (Fig. 4B).

### Expression levels and diagnostic potential of crucial hub genes

To ensure the accuracy of the screened hub genes, we utilized the GSE140713, GSE77939, GSE2171, GSE33463, GSE703, and GSE19617 datasets for validation. ISG15 was significantly upregulated in three datasets, but not in GSE33463, GSE2171, and GSE19617. IFI27 was upregulated significantly in both HIV-1 or PAH (all *P* < 0.05, Fig. 4C). In addition, we performed ROC analysis to assess the diagnostic potential of ISG15 and IFI27. ISG15 demonstrated good diagnostic potential in the remaining three datasets, with the exception of the GSE33463, GSE2171, and GSE19617 datasets (Fig. 4D). IFI27 demonstrated robust diagnostic potential in all six datasets, particularly in the GSE703 dataset, where it exhibited nearly perfect diagnostic capability with an AUC value of 0.940. Additionally, we observed an increasing trend in the expression of ISG15 and IFI27 among HIV patients on subgroup analysis, regardless of whether they received ART or not (Fig. 4C, Fig. S6A,B). In HIV patients during the early stages of ART treatment, there was a significant decrease in the expression of ISG15 and IFI27 compared to the control group (*P* < 0.05, Fig. S6A,B). Similarly, within distinct PAH subgroups, both IPAH and SPAH patients exhibited increasing trends in the expression of ISG15 and IFI27. However, consistent significant differences in the expression of ISG15 and IFI27 between IPAH and SPAH patients were not observed (Fig. S6C-F).

### Construction of hub genes PPI network and GSEA

**Figure 5:**
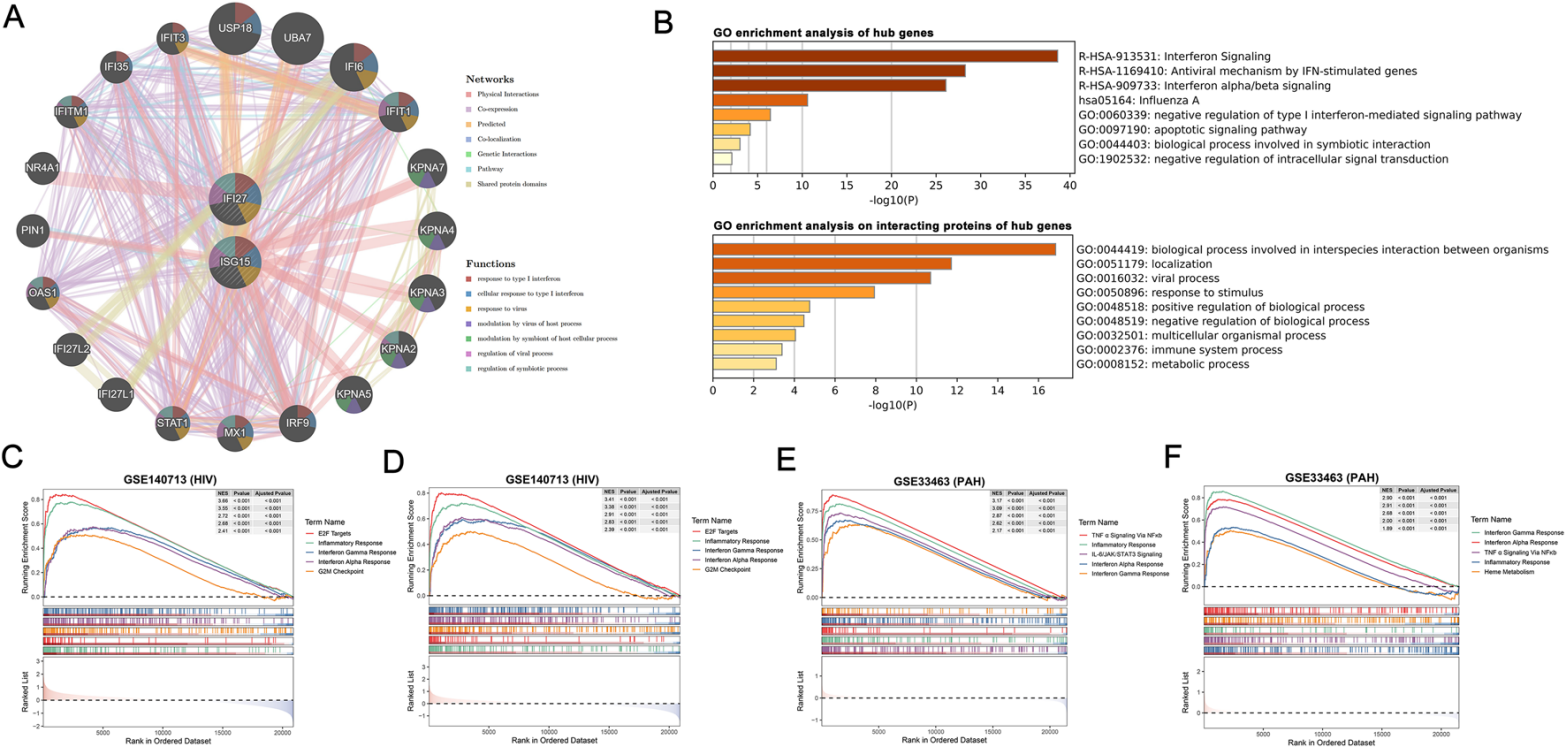
PPI network and Gene set enrichment analysis (GSEA) of hub genes. (**A**) PPI network of hub genes and their interacting proteins. (**B**) GO enrichment analysis of hub genes and their interacting proteins. (**C**) GSEA of the top 5 enriched pathways in HIV-1 patients with high ISG15 expression from the GSE140713 dataset. (**D**) GSEA of the top 5 enriched pathways in HIV-1 patients with high IFI27 expression from the GSE140713 dataset. (**E**) GSEA of the top 5 enriched pathways in PAH patients with high ISG15 expression from the GSE33463 dataset. (**F**) GSEA of the top 5 enriched pathways in PAH patients with high IFI27 expression from the GSE33463 dataset.* GO* gene ontology, *NES* Normalized Enrichment Score, *PAH* pulmonary arterial hypertension.

We performed protein–protein interaction analysis centered on the crucial hub genes through the GeneMania database, and the outcomes demonstrated that the majority of proteins associated with ISG15 and IFI27 were IFN-induced proteins and karyopherin-α (KPNA) proteins (Fig. 5A). Hub genes and their co-expressed genes predominantly participated in the type 1 IFN signaling pathway and the antiviral immune response pathway. Furthermore, we performed GO functional and pathway enrichment analysis on these genes using the metascape database. Hub genes were found to be related to interferon signaling, the antiviral mechanism by IFN-stimulated genes, and the biological process involved in interspecies interactions between organisms and the viral process (Fig. 5B).

GSEA was utilized on hallmark gene sets to identify functional enrichments in high and low hub genes expressions across the GSE140713 and GSE33463 datasets. The results of GSEA showed that among the top five differentially expressed upregulated enriched pathways associated with high hub genes expression, all were linked to responses involving type I IFN, the IFN gamma response, and the inflammatory response (all adjusted *P* < 0.05, Fig. 5C–F).

### Immune infiltration analysis

**Figure 6:**
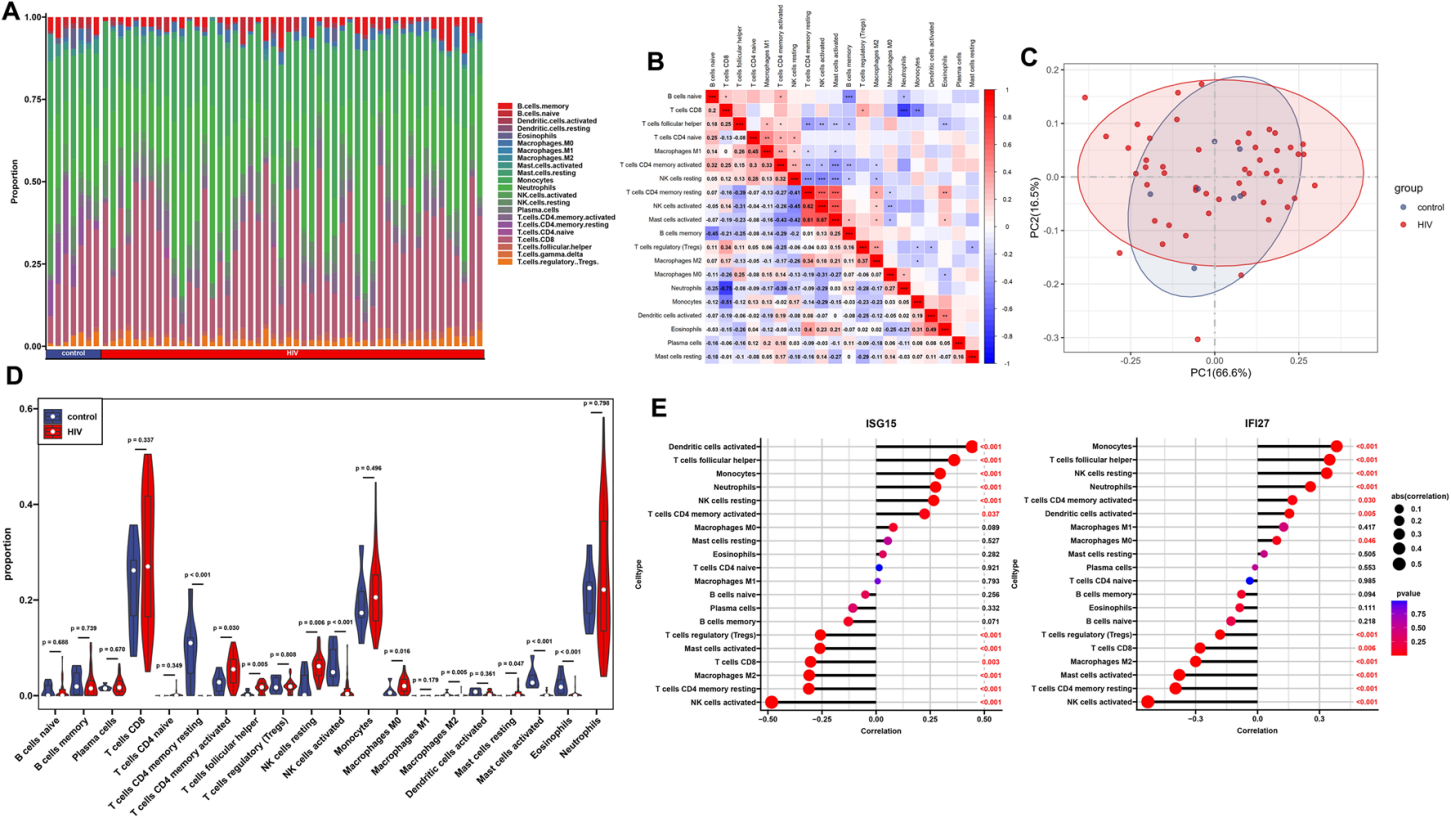
Immune infiltration analysis by CIBERSORT for the GSE140713 (HIV) dataset. (**A**) The proportion of 22 immune cells in various samples. (**B**) Co-expression analysis of 22 infiltrating immune cells in HIV-1 samples. (**C**) The PCA cluster plot of immune cell infiltration in HIV-1 and control samples. (**D**) The violin plot showed the infiltration of immune cells in HIV-1 and control samples. (**E**) Correlation analysis between hub genes expression and the proportion of immune cells in HIV-1 samples. *PCA* principal component analysis; *P < 0.05; **P < 0.01; ***P < 0.001.

**Figure 7:**
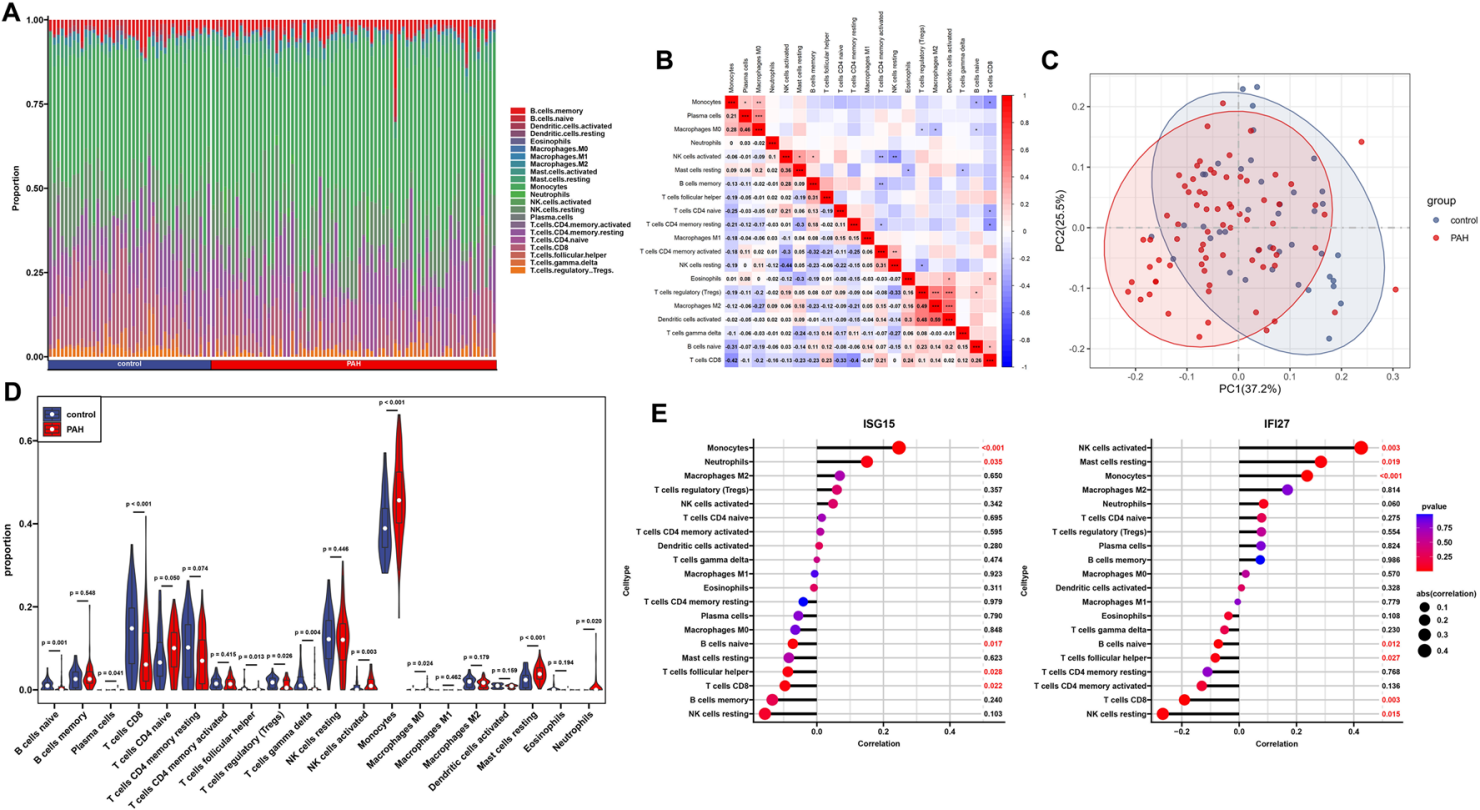
Immune infiltration analysis by CIBERSORT for the GSE33463 (PAH) dataset. (**A**) The proportion of 22 immune cells in various samples. (**B**) Co-expression analysis of 22 infiltrating immune cells in PAH samples. (**C**) The PCA cluster plot of immune cell infiltration in PAH and control samples. (**D**) The violin plot showed the infiltration of immune cells in PAH and control samples. (**E**) Correlation analysis between hub genes expression and the proportion of immune cells in PAH samples. *PCA* principal component analysis, *PAH* pulmonary arterial hypertension; *P < 0.05; **P < 0.01; ***P < 0.001.

To assess the immune cell immersion between HIV-1/PAH patients and the control group, we analyzed the immune infiltration for the GSE140713 and GSE33463 gene expression data separately. The cumulative histogram shows the proportions of 20 different types of immune cells (Figs. 6A, 7A). We then further analyzed the correlations between 20 different types of immune cells (Figs. 6B, 7B). In the HIV-1 dataset, we found significant positive correlations between activated NK cells and activated memory CD4 T cells (r = 0.62); activated mast cells and activated memory CD4 T cells (r = 0.61); and activated mast cells and activated NK cells (r = 0.67). There was a significant negative correlation between neutrophils and CD8 T cells (r =  − 0.75). In the PAH dataset, we found significant positive correlations between M2 and regulatory T cells (r = 0.49), activated DCs and regulatory T cells (r = 0.48); and activated DCs and M2 (r = 0.58).

There was a significant reduction in the infiltration of M0, resting mast cells, and eosinophils in both the HIV-1 and PAH conditions (Figs. 6C, 7C). Individuals in the control group and the HIV-1/PAH group could be separated into two distinct groups by PCA based on 20 immune cell subpopulations (Figs. 6D, 7D). Additionally, in the HIV-1 group, a correlation analysis of different types of immune cells with hub genes demonstrated a positive correlation between hub genes and specific cell populations, including T follicular helper cells, resting NK cells, activated DCs, monocytes, and neutrophils (*P* < 0.05, Fig. 6E). Within the PAH group, the hub genes showed a positive correlation exclusively with monocytes (*P* < 0.05, Fig. 7E).

### Single-cell analysis for hub genes

**Figure 8:**
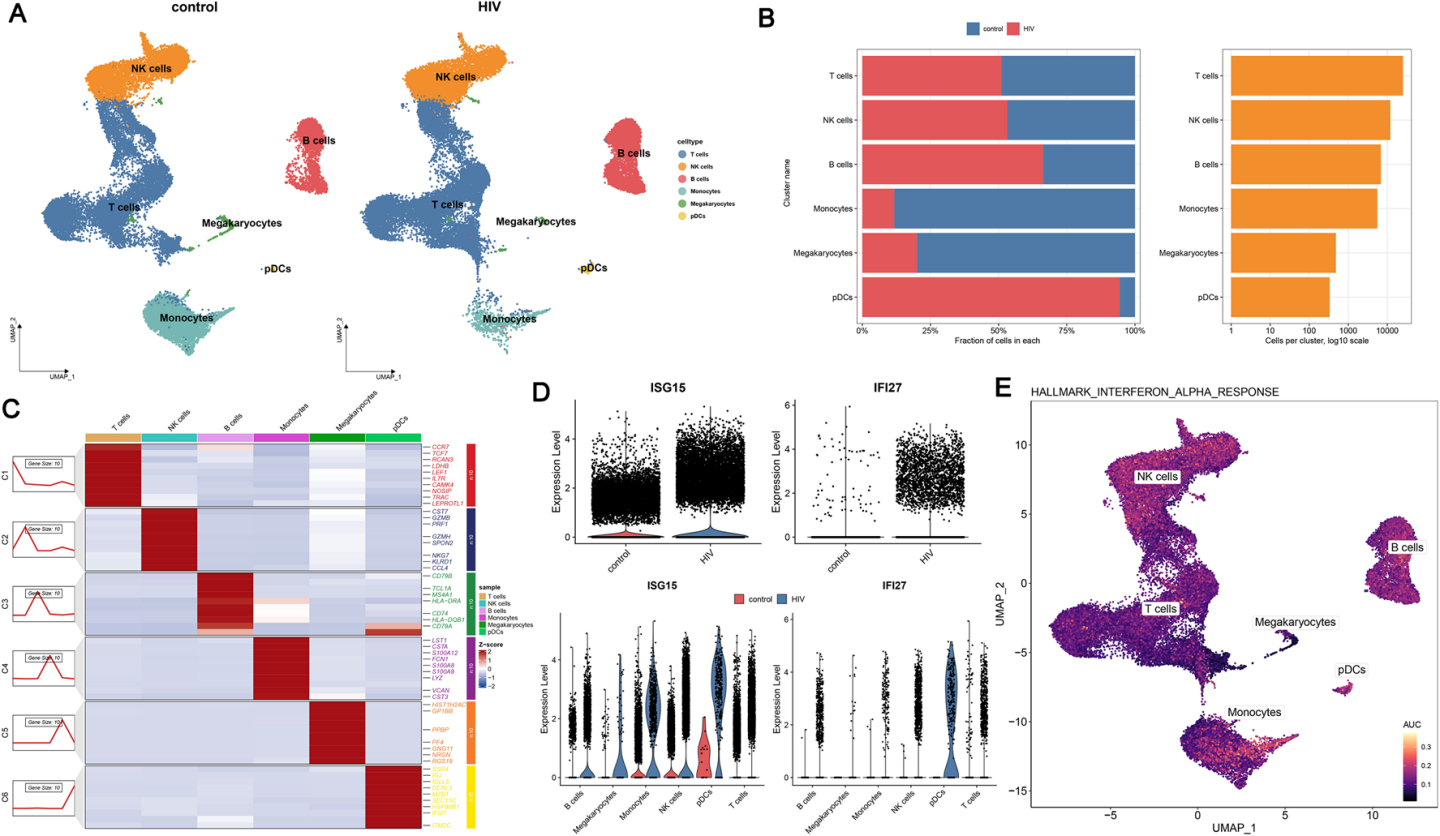
Single-cell Dataset Clustering Analysis. (**A**) The UMAP plots showed the results of applying the marker genes to annotate cells. (**B**) The left-side bar showed the percentage of each cell type in healthy and HIV-infected donors. The right-side bar showed the number of each type of cell. (**C**) Heatmap of the expression of the hallmark genes by different cell clusters. (**D**) The combined violin plot showed the expression levels of ISG15 and IFI27 across various cell clusters at the top and compares their expression in healthy and HIV-infected donors at the bottom. (**E**) The UMAP plot showed the AUC scores of interferon alpha response pathways for each cell. *UMAP* uniform manifold approximation and projection, *AUC* area under the curve.

In order to further investigate the immune microenvironment of peripheral blood, we utilized single-cell RNA sequencing data to explore the expression of hub genes across different cell types. After quality control (Fig. S7), integration (Fig. S8A), dimensionality reduction, and clustering (Fig. S8B,C), a total of 50,524 cells (healthy donors: 25,596, HIV-1 infected donors: 24,928) were obtained and clustered into 15 clusters. We annotated the cells based on marker genes and classified the 15 clusters into six distinct cell populations: encompassing T cells (25,302), NK cells (11,982), B cells (6851), monocytes (5569), megakaryocytes (484), and pDCs (336) (Fig. 8A). We conducted a comparative analysis of the quantities of various cell subpopulations between the two groups and observed significant reductions in monocytes and megakaryocytes, while B cells and pDCs showed significant increases in individuals infected with HIV-1 compared to the healthy controls (Fig. 8B). Cluster marker genes were identified via the FindAllMarker function in Seurat (Fig. 8C). Meanwhile, we observed elevated expression levels of hub genes in the HIV-1 group (Fig. 8D), which is consistent with the previous transcriptomic findings. To further assess the expression patterns of hub genes across various cell subsets, we performed an analysis of their expression within each cell subpopulation, and the results showed that ISG15 was highly expressed by monocytes, NK cells, and pDCs, while IFI27 was highly expressed exclusively in pDCs. The results were similar to those obtained from CIBERSORT.

We also calculated the area under the curve (AUC) scores for the crucial hallmark pathway (type I IFN response) associated with each cell's hub genes. As expected, the pathway score for the type I IFNresponse was elevated in monocytes, NK cells, and pDCs, particularly monocytes (Fig. 8E). In other words, monocytes are pivotal cells involved in the type I IFN response pathway.

## Discussions

PAH is a complex and incurable disease whose mechanisms involve a variety of inflammatory responses^25^. As one of the most serious complications of HIV/AIDS, PAH has a high mortality rate and is progressively increasing among HIV-1 patients^26^, which has seriously affected the prognosis of HIV-1 patients. However, there is currently insufficient concrete evidence regarding the specific association between HIV-1 and PAH. There also appears to be limited research exploring the susceptibility relationship to PAH in HIV-1 patients at the genetic level. Consequently, identifying potential genes and mechanisms related to both HIV-1 and PAH may offer new perspectives to uncover the underlying connection. In this study, through bioinformatics analysis, we have identified hub genes and identified crucial pathways between HIV-1 and PAH.

In this study, our objective was to explore the shared molecular mechanisms between HIV-1 and PAH. After conducting a cross-analysis across different datasets, ISG15 and IFI27 were identified as pivotal hub genes between HIV-1 and PAH that are highly associated with the type I IFN signaling pathway. ISG15 is a ubiquitin-like protein that becomes attached to target proteins via ISGylation, a process mediated by E1, E2, and E3 enzymes^27^. This modification is prompted by type I IFNs, leading to increased expression when the IFN signaling pathway is activated^28^. ISG15 is mainly secreted by neutrophils, monocytes, and lymphocytes, and can act on T and NK cells to induce IFNγ production and play a critical role in the IFN-induced innate immune response^29–32^. IFI27, also known as ISG12a (IFN-stimulated gene 12a), belongs to the IFI6/IFI27 family^33^ and, similar to ISG15, is a type I IFN-inducible protein involved in various biological processes, including cell apoptosis and antiviral activity induced by type I IFNs^34^. Notably, previous studies have reported that ISG15 and IFI27 are highly correlated with HIV-1 infection^35–37^. Additionally, in the subgroup analysis of HIV-1, we observed reduced expression of ISG15 and IFI27 in immune responders compared to ART-naive individuals and immune non-responders. Although no significant differences were observed, this is consistent with previous studies^38,39^. The prolonged inflammatory response and altered immune processes over time contribute to the development of PAH^40^. The inflammation induced by the elevated expression of ISG15 and IFI27 may serve as the foundation for the occurrence of PAH, suggesting that immunofailers may have a higher likelihood of developing PAH. Despite increasing evidence confirming a link between type I IFN and PAH^41^, there are currently no reported studies on the associations between ISG15, IFI27, and PAH. In this study, ISG15 and IFI27 demonstrated promising diagnostic potential in the PAH dataset, and they may serve as potential biomarkers for PAH.

IFNα is a cytokine of the innate immune system, which is an important mediator of inflammation^42^. HIV-1 infection typically results in the production of proinflammatory cytokines (IL-6) and IFNα. As one of the earliest stages of host immune defense, the innate immune response is mediated by IFNα and operates by upregulating the expression of IFN-stimulated genes with diverse anti-HIV properties^43^, thereby inhibiting the replication of HIV, especially in acute HIV-1 infection^44^. However, during chronic infections, the direct link between persistent IFNα signal transmission and HIV-1-induced immunopathogenesis remains uncertain^45^, but sustained activation of IFN signaling still^46^. Although type I IFN levels are reduced under combination ART (cART), IFN-stimulated genes (ISGs) are still upregulated in PBMC or lymphoid organs^46,47^, which is consistent with the findings in this study.

PAH consists of several subtypes, including idiopathic (IPAH), familial (FPAH), and secondary PAH (SPAH). However, research derived from a meta-analysis of the genome-wide blood expression profiles in both IPAH and SPAH revealed the existence of common immunologic mechanisms^48^. Furthermore, compared to a healthy control, these DEGs are enriched in biological processes such as the type I IFN response, antiviral immune response, and Toll-like receptor signaling, which is consistent with the shared DEGs enrichment results in this study. Several studies have demonstrated that IFN response is highly active in PAH^49,50^. Human pulmonary vascular cells are sensitive to type I IFN, and high levels of IFNα stimulation release IFNγ inducible protein 10 (IP10) and endothelin-1 (ET-1) through inducing TNFα-primed human pulmonary artery smooth muscle cells (HPASMCs)^41^. IP10 and ET-1 serve as pivotal mediators in the pathogenesis of PAH. Moreover, HIV-1 infection leads to a reduction in tight junction proteins that preserve the integrity of the pulmonary endothelial barrier, thereby directly damaging endocrin cells^51^. This may be a trigger point for endocranial dysfunction and the pathogenic mechanism of PAH^52^. PAH has a high incidence among HIV-1 patients, implying a potential role of HIV-1 in promoting PAH pathogenesis. Consequently, we speculated that type I IFN plays a pivotal role in the pathogenic occurrence of HIV-related PAH. Despite recent advances in pharmacotherapy for individuals with PAH, treatments are mostly of limited effectiveness^53^. Considering the modulation of the IFN pathway as a potential therapeutic target for treating PAH, this holds promising implications for guiding future treatments, prevention strategies, and the development of vaccines for individuals with concurrent HIV-1 and PAH.

Meanwhile, we have observed that the reduction in monocyte numbers in HIV patients may be associated with compromised immune function. Monocytes, as primary participants in the innate immune system, play a crucial role in antiviral infections through the IFN signaling pathway^54^. Monocytes serve as target cells for HIV-1 infection, and monocytes release inflammatory factors and differentiate upon HIV-1 infection, stimulated by the IFN signaling pathway^55^. Furthermore, based on the results of immune infiltration analysis, a significant positive correlation between ISG15 and IFI27 expression and monocytes has been observed in both HIV-1 and PAH, and it could therefore be inferred that the upregulation of ISG15 and IFI27 promotes monocyte activation and differentiation into pDCs during migration by IFN stimulation^56^. This explains the notable enrichment of type I IFN within the pDCs, as shown in the scRNA-seq results.

We used integrated bioinformatics methods such as the WGCNA algorithm to identify common hub genes and pathways in HIV-1 and PAH, which further elucidate the pathogenesis of both. However, there are certain limitations of our study.Firstly, the limited sample size employed for the bioinformatics analyses could potentially impact the robustness of our findings. Our data is derived from multiple datasets, and there may be some heterogeneity among the different datasets. Secondly, we lack in vitro experiments to further validate our results. Furthermore, our study is confined to the transcriptomic level, and the integration of multi-omics data will be essential to validate our conclusions in the future.

In summary, our study utilized a bioinformatics analysis to illustrate the possible mechanism of PAH secondary to HIV-1 and showed that the heightened IFN response in HIV-1 might be a crucial susceptibility factor for PAH, with monocytes being pivotal cells involved in the type I IFN response pathway. This provides potential new insights for further investigating the molecular mechanisms connecting HIV-1 and PAH.

### Supplementary Information

Supplementary Figure 1.

Supplementary Figure 2.

Supplementary Figure 3.

Supplementary Figure 4.

Supplementary Figure 5.

Supplementary Figure 6.

Supplementary Figure 7.

Supplementary Figure 8.

Supplementary Legends.

## Data Availability

The datasets presented in this study can be found in online repositories. The names of the repositories and accession numbers can be found in the article or supplementary material.
