# Supplementary figures and images for: Identification of the shared hub gene signatures and molecular mechanisms between HIV-1 and pulmonary arterial hypertension
Source: Sci Rep. 2024 Mar 25;14:7048. doi: 10.1038/s41598-024-55645-x (PMC10963360; doi:10.1038/s41598-024-55645-x)

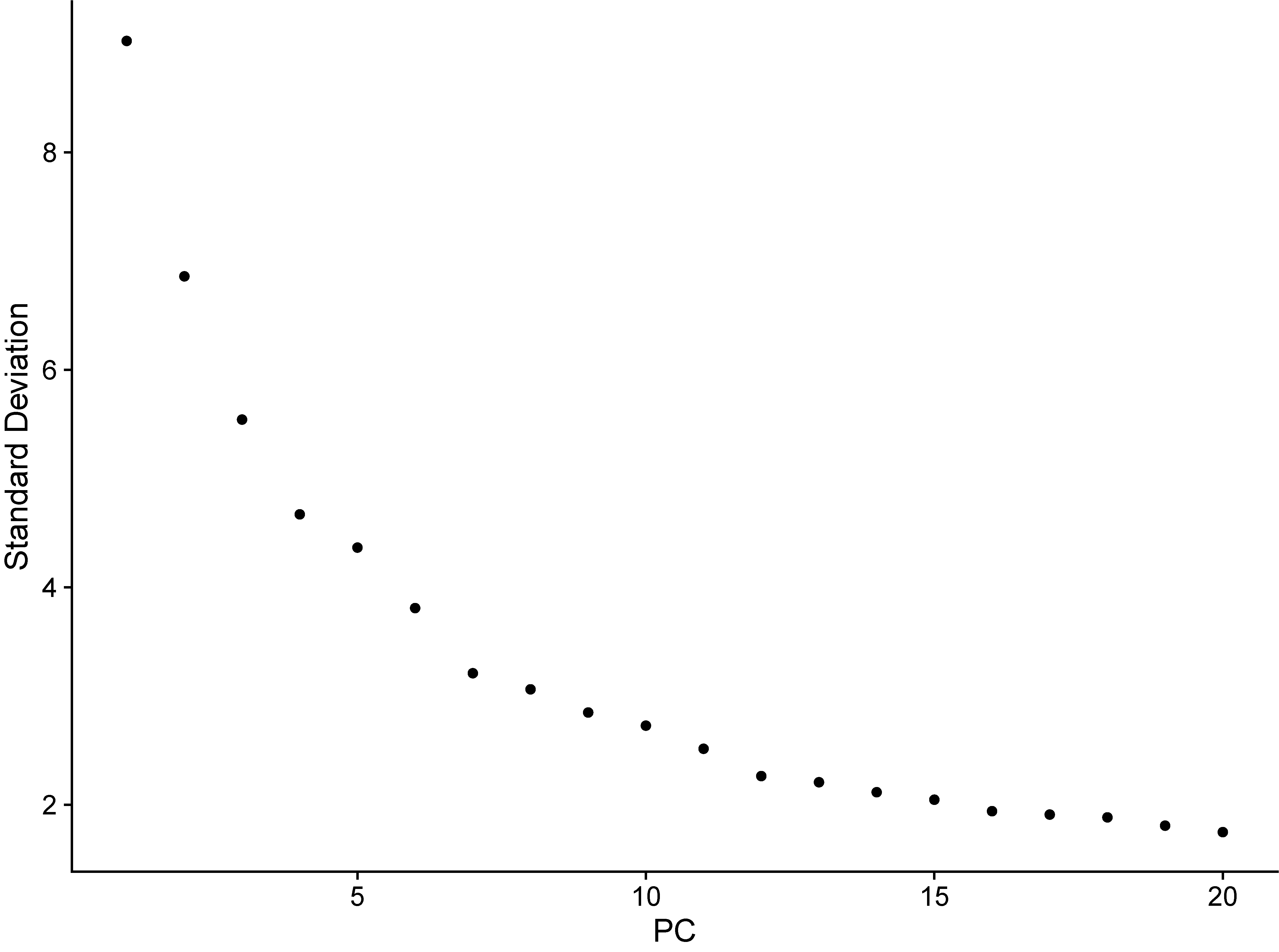

Supplement: Supplementary file 1 — Supplementary Figure 1. [file 41598_2024_55645_MOESM1_ESM.tif]

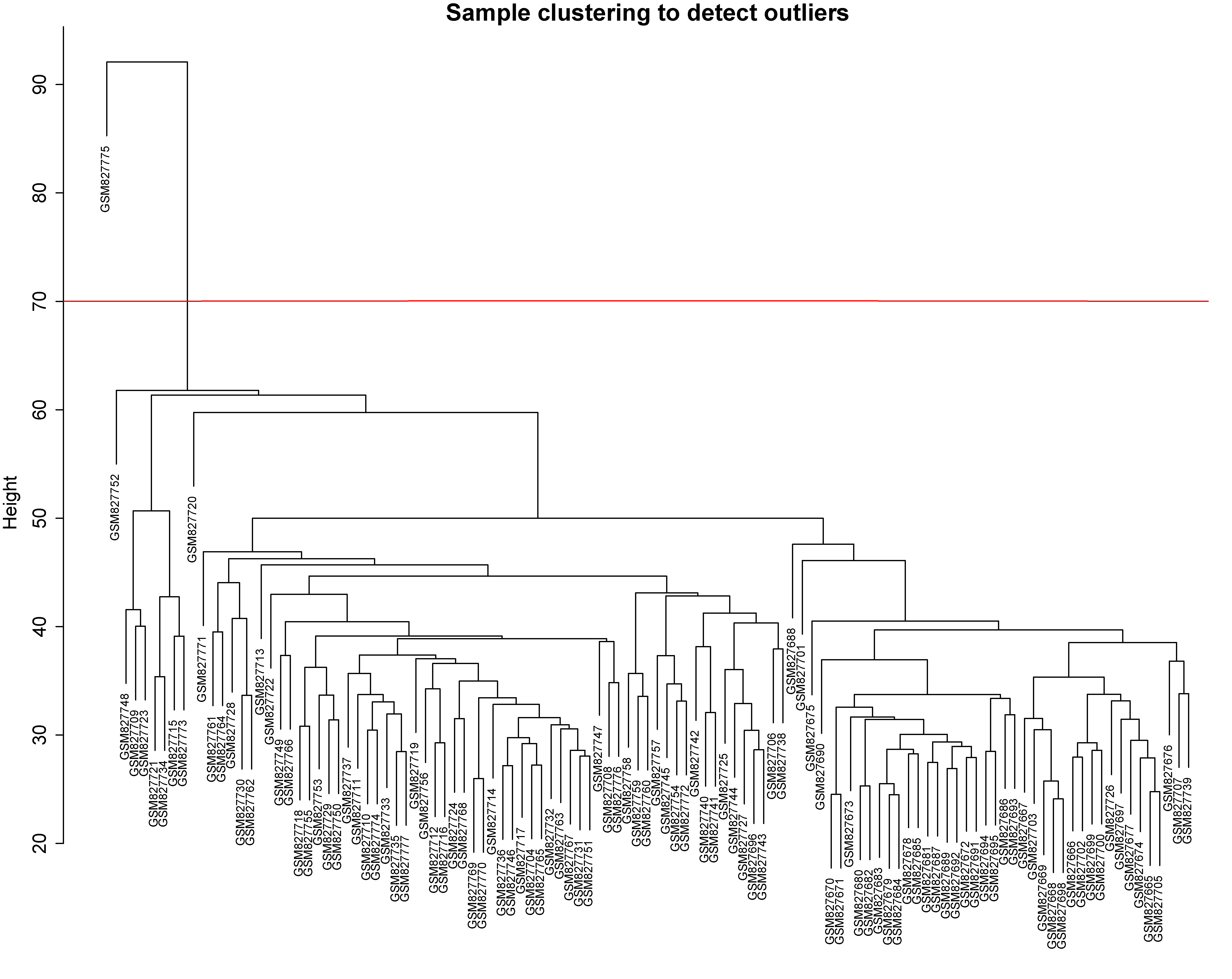

Supplement: Supplementary file 4 — Supplementary Figure 4. [file 41598_2024_55645_MOESM4_ESM.tif]

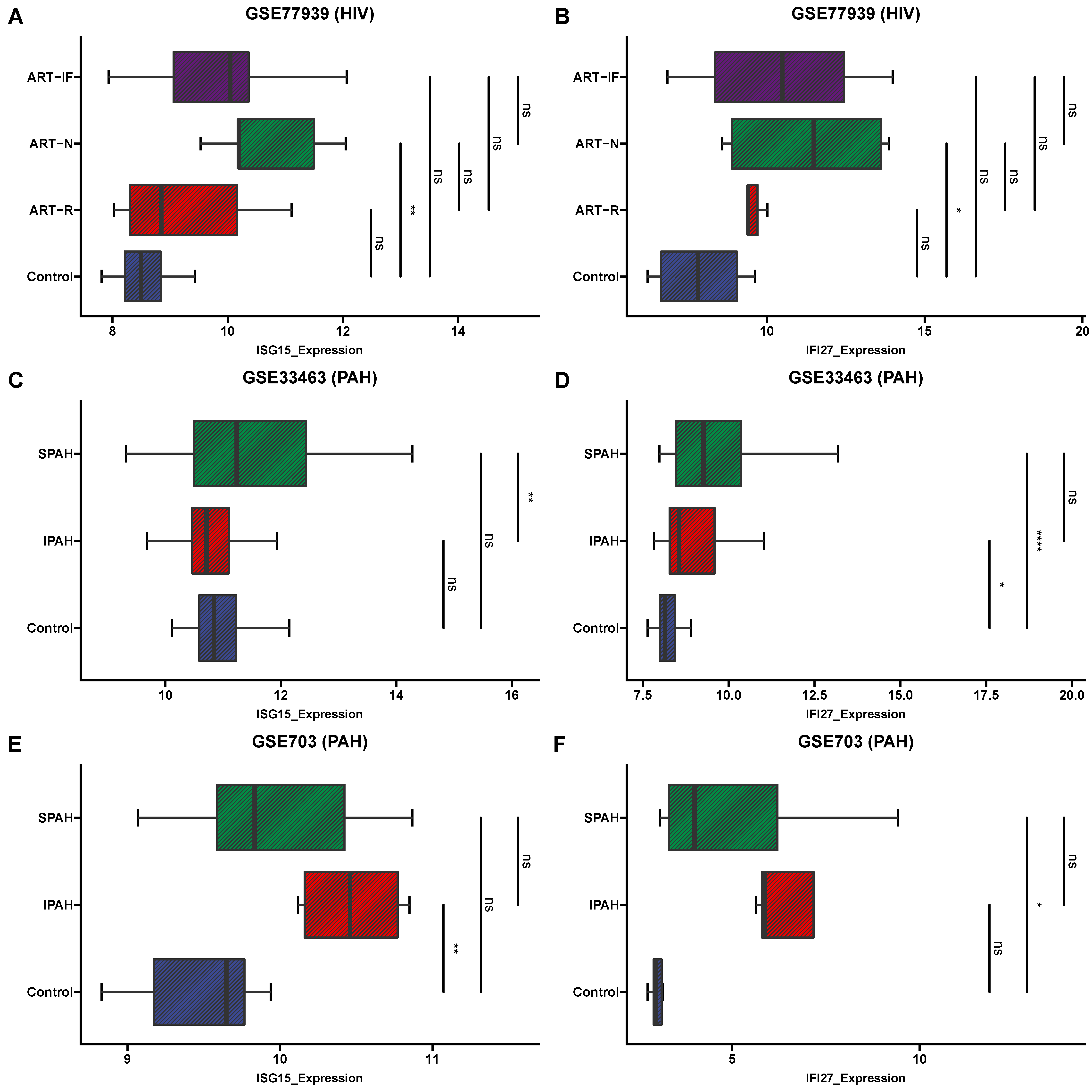

Supplement: Supplementary file 6 — Supplementary Figure 6. [file 41598_2024_55645_MOESM6_ESM.tif]
