# Supplementary material for: Identification of the shared hub gene signatures and molecular mechanisms between HIV-1 and pulmonary arterial hypertension
Source: Sci Rep. 2024 Mar 25;14:7048. doi: 10.1038/s41598-024-55645-x (PMC10963360; doi:10.1038/s41598-024-55645-x)
Supplement: Supplementary file 9 — Supplementary Legends. [file 41598_2024_55645_MOESM9_ESM.docx]

**Supplementary Legends**

**Supplementary Fig. S1. The elbow plot of the optimal number of principal components.**

**Supplementary Fig. S2. The box plot of gene expression level in different datasets after background correction and normalization.** PAH, pulmonary arterial hypertension.

**Supplementary Fig. S3.** **(A)** Sample dendrogram and trait heatmap in GSE140713 (HIV). **(B)** Sample dendrogram and trait heatmap in GSE33463 (PAH). **(C)** The cluster dendrogram of co-expression genes in GSE140713 (HIV). **(D)** The cluster dendrogram of co-expression genes in GSE33463 (PAH). PAH, pulmonary arterial hypertension.

**Supplementary Fig. S4. The sample tree diagram shows the clustering of 113 samples in the GSE33463 (PAH) dataset, detecting one outlier sample (GSM827775).** PAH, pulmonary arterial hypertension.

**Supplementary Fig. S5. The PCA plot shows clusters of samples based on similarity for the GSE77939 (HIV) dataset and the GSE703 (PAH) dataset.** PCA, Principal component analysis; PAH, pulmonary arterial hypertension.

**Supplementary Fig. S6. Evaluation of hub genes using different external datasets.** ART-IF: antiretroviral therapy immunolgical failure (Individuals undergoing ART for at least one year, exhibiting an undetectable viral load (<40 copies/mL), and maintaining CD4 T cell counts below 250 cells/μL); ART-N: antiretroviral therapy naive (ART-naive individuals with a CD4 T cell count exceeding 200 cells/μL); ART-R: antiretroviral therapy responder (Individuals undergoing ART for at least one year, showing a positive response to the treatment by exhibiting an increase in CD4 T cell counts of over 150 cells/μL, with current CD4 counts above 250 cells/μL); IPAH: idiopathic pulmonary arterial hypertension; SPHA: secondary pulmonary arterial hypertension. ^*^P < 0.05; ^**^P < 0.01; ^****^ P < 0.0001.

**Supplementary Fig. S7. Single-cell RNA-seq data quality control. (A)** The violin plots of gene counts (nFeature_RNA), UMI counts (nCount_RNA), and the percentage of mitochondrial gene counts (pecent_mito) before filtering. **(B)** The left-side scatterplot shows the correlation between nFeature_RNA and nFeature RNA**.** The right-side scatterplot shows the correlation between nCount RNA and pecent_mito. **(C)** The violin plots of nFeature_RNA, nCount_RNA, and pecent_mito after filtering. **(D)** The cell cycle score was calculated using the CellCycleScoring function in Seurat.

**Supplementary Fig. S8. Single-cell Dataset Clustering Annotation. (A)** The left-side UMAP plot shows the distribution of cells from healthy and HIV-infected donors after undergoing CCA integration. The right-side UMAP plot shows the distribution of cells from healthy and HIV-infected donors before undergoing CCA integration. **(B)** Cluster tree plot of the number of clusters under different resolution conditions. **(C)** The UMAP plot of integration data with 15 clusters (resolution = 0.8). **(D)** The stack violin plot shows the cell marker gene expression in each cell cluster. CCA, canonical correlation analysis; UMAP, uniform manifold approximation and projection.
